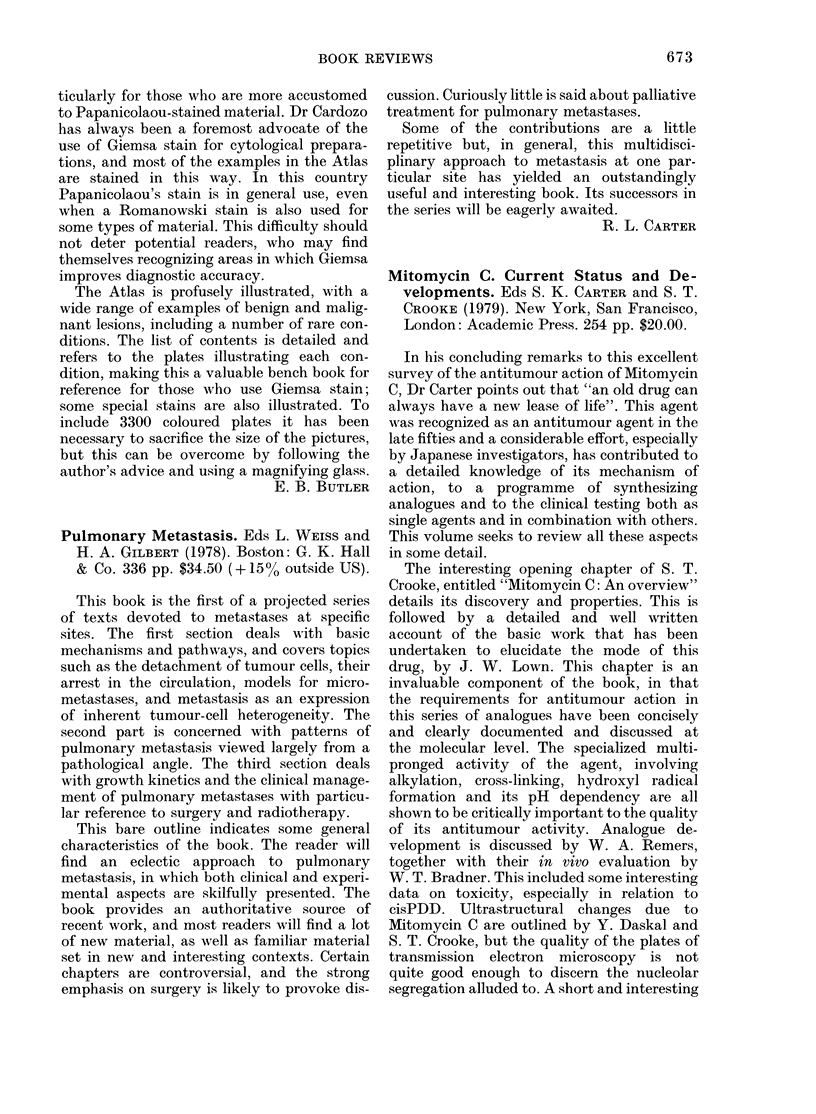# Pulmonary Metastasis

**Published:** 1980-04

**Authors:** R. L. Carter


**Pulmonary Metastasis.** Eds L. Weiss and H. A. Gilbert (1978). Boston: G. K. Hall & Co. 336 pp. $34.50 (+15% outside US).

This book is the first of a projected series of texts devoted to metastases at specific sites. The first section deals with basic mechanisms and pathways, and covers topics such as the detachment of tumour cells, their arrest in the circulation, models for micrometastases, and metastasis as an expression of inherent tumour-cell heterogeneity. The second part is concerned with patterns of pulmonary metastasis viewed largely from a pathological angle. The third section deals with growth kinetics and the clinical management of pulmonary metastases with particular reference to surgery and radiotherapy.

This bare outline indicates some general characteristics of the book. The reader will find an eclectic approach to pulmonary metastasis, in which both clinical and experimental aspects are skilfully presented. The book provides an authoritative source of recent work, and most readers will find a lot of new material, as well as familiar material set in new and interesting contexts. Certain chapters are controversial, and the strong emphasis on surgery is likely to provoke discussion. Curiously little is said about palliative treatment for pulmonary metastases.

Some of the contributions are a little repetitive but, in general, this multidisciplinary approach to metastasis at one particular site has yielded an outstandingly useful and interesting book. Its successors in the series will be eagerly awaited.

R. L. Carter